# From profiles to precision: incorporating the Psychosocial Assessment Tool (PAT) and the Strengths and Difficulties Questionnaire (SDQ) to inform treatment planning in outpatient psychiatry

**DOI:** 10.1007/s00787-026-02983-y

**Published:** 2026-04-24

**Authors:** Shirel Dorman-Ilan, Yaara Sadeh, Naama de la Fontaine, Noa Rubin, Tamar Silberg, Doron Gothelf, Shlomit Tsafrir

**Affiliations:** 1 The Edmond and Lily Safra Children’s Hospital, Sheba Medical Center, Ramat Gan, Israel; 2 School of Psychological Sciences, Tel Aviv University, Tel Aviv, Israel; 3 Department of Pediatric Rehabilitation, the Edmond and Lily Safra Children’s Hospital, Chaim Sheba, Ramat Gan, Israel; 4 School of Social Work, University of Haifa, Haifa, Israel; 5 Child Study Center, Yale School of Medicine, New Haven, CT USA; 6 Department of Psychology, Bar Ilan University, Ramat Gan, Israel; 7 The Gray Faculty of Medical and Health Sciences, Tel Aviv University, Tel Aviv, Israel; 8 Sagol School of Neuroscience, Tel Aviv University, Tel Aviv, Israel; 9 The Donald Cohen Child and Adolescent Psychiatry Department, Jerusalem Mental Health Center, Eitanim Psychiatric Hospital, Jerusalem, Israel; 10 Hebrew University, Hadassah School of Medicine, Jerusalem, Israel

**Keywords:** Psychosocial screening, Child psychiatry, Triage, PAT, SDQ, Treatment planning

## Abstract

This study aimed to evaluate the feasibility of incorporating an integrative screening procedure comprised of the Psychosocial Assessment Tool (PAT), Strengths and Difficulties Questionnaire (SDQ), and The Child PTSD Symptom Scale (CPSS-5) (part A) into the initial referral process at a public outpatient child and adolescent psychiatry clinic. We aimed to (1) evaluate the feasibility of the screening procedure; (2) identify distinct psychosocial profiles through latent profile analysis (LPA); and (3) determine whether these unique profiles correlate with varying symptom severity levels to enhance treatment precision. Data from 901 families referred to a child and adolescent psychiatry clinic between January 2024 and January 2025 were analyzed. Parents completed the PAT, SDQ and CPSS-A online prior to intake. LPA was used to identify psychosocial profiles. A pre-post comparison assessed whether implementation of screening increased rates of comprehensive care (psychiatric and para-medical treatment). Screening completion was high (95.7%), indicating strong feasibility. The LPA revealed three distinct profiles: Child Focused Profile (79.1%), Low Social Support Profile (12.5%), and High Parental Stress Profile (8.5%). These profiles differed in trauma exposure, family structure, and SDQ symptom patterns. Following implementation, a significantly greater proportion of patients received comprehensive care compared to the previous year (82.1% vs. 77.5%, *p* = .012). Integrating the PAT, SDQ and CPSS-A at intake is feasible and facilitates meaningful psychosocial profiling that may support triage and tailored interventions. Profile-informed care has the potential to enhance treatment precision and resource allocation in overstretched mental health systems.

## Introduction

 Current mental health systems struggle with promptly assessing, diagnosing, and treating children, typically delaying care until after extended waiting periods [1, 2]. Current uniform waitlists stem from a reactive model where comprehensive assessments occur only after families endure long waiting times, without considering critical risk factors, such as parental distress or environmental instability. This structural shortcoming has cascading consequences, influencing treatment engagement and even discouraging families from seeking help altogether [3, 4].

Research indicates that prolonged waiting periods are associated with significant exacerbation of symptoms. A survey of approximately 14,000 young people found that more than a quarter had attempted suicide while waiting for mental health support [5]. Similarly, other studies have reported an increase in self-harm behaviors among young adults while on waiting lists. Furthermore, prolonged wait times undermine trust in the mental health system and contribute to premature treatment dropout. These patterns not only delay access to evidence-based interventions but also disrupt treatment continuity and diminish the likelihood of positive outcomes [4, 6].

Another significant concern is the issue of misdirected referrals, which compromises the precision of treatment [7], such as, reported high rejection rates for children and adolescent referrals from physicians, attributing this to inadequate referral processes. Specifically, re-referrals constituted more than a third of all referrals (35.9%), indicating that the referrals may have been inaccurate or not tailored to the individual’s needs or capacity. Misdiagnosis or inappropriate treatment allocation often leads to family frustration, eroding trust in healthcare providers and reducing treatment adherence [8, 9].

In recent years, stratified care models have gained increasing attention as an effective framework for addressing these systemic gaps. Stratified care emphasizes matching families to the least intensive, yet sufficiently effective, level of intervention based on empirically assessed risk and need, rather than relying on uniform waitlists or clinician judgment alone. Such models improve resource allocation, reduce unnecessary delays, and enhance treatment precision, an approach directly aligned with the aims of the current study [10–12].

To effectively address these systemic challenges, incorporating validated tools, such as the Strengths and Difficulties Questionnaire [13], the Psychosocial Assessment Tool [14], and the Child PTSD Symptom Scale (CPSS-A) [15], into referral processes presents a promising approach. This strategy may facilitate risk stratification and enable individually tailored treatment planning at first contact. The PAT, initially designed to assess psychosocial risk factors among families of children diagnosed with cancer [14], has since been applied in diverse medical settings, reliably providing a systematic evaluation of psychosocial aspects of patient care, which enhances the ability to tailor care pathways based on family-specific needs [16–18]. PAT scores map onto the three-tier Pediatric Psychosocial Preventative Health Model (PPPHM), which categorizes families into low, moderate, or high psychosocial risk. This framework helps identify the intensity of support required, from routine monitoring for low-risk families to targeted or intensive intervention for those facing more substantial or escalating psychosocial difficulties. Likewise, The SDQ has demonstrated utility as a brief screening tool for early triage in child and adolescent mental health services (CAMHS). SDQ scores have been shown to align with clinician-rated severity and referral appropriateness in pre-admission CAMHS settings [19] and to demonstrate good agreement with comprehensive diagnostic measures [20]. These findings support the SDQ’s utility for first-stage risk identification and treatment planning in outpatient care [21, 22]. In addition, Screening for exposure to potentially traumatic events is increasingly recognized as a critical component of initial mental health assessments, as it enables early identification of youth who may require further trauma-focused evaluation [23, 24]. Consistent with this recommendation, the CPSS-A was used in the present study as a brief front-line screening tool to identify a clinically relevant index trauma warranting further assessment.

To our knowledge, only a single study reported using the PAT as a screening tool among 87 families during their initial psychiatric evaluation [25]. All families received case-management support appropriate to their assigned risk level. For those reporting persistent stressors or escalating distress, the intensity of case-management services increased, with more frequent and longer-term intervention (typically lasting 1–2 months). Analysis of the PAT responses indicated three predominant categories of psychosocial stressors. First, child problems (i.e., highly symptomatic patient concerns at the onset of treatment) represented the most frequent area of need among most families (68%). Second, parent/caregiver stress reactions (e.g., parental mental health concerns) emerged as substantial needs among families experiencing chronic or escalating distress and high-risk factors (64%). Third, family structure and resources (e.g., financial strain, limited social support) were noted as key areas of need among families encountering acute distress with identifiable risk factors (55%). Results showed that families who completed the PAT and participated in intensive case management intervention showed higher treatment adherence. Although significant, the PAT was administered only after a prolonged wait-list time, thus having no effect on initial triage and intake procedures. Furthermore, the relatively small sample size (*N* = 87) warrants further assessment.

An ecological approach to mental health care requires systems to move beyond first-come-first-served models and implement data-driven triage processes that comprehensively assess risk factors, protective factors, symptoms, and family context from the initial point of contact. Thus, the aims of the current study were threefold. Our first aim was to evaluate the feasibility of using the PAT, SDQ and CPSS-A as online screening tools completed by parents prior to their child’s initial assessment at an outpatient child and adolescent psychiatry clinic. Our second aim was to characterize the psychosocial profiles and symptom patterns of referred children to identify distinct subgroups within this clinical population, based on the PAT, SDQ and CPSS via exploratory latent class analysis (LCA). While the analysis was data-driven, we hypothesized that: (1) in most cases, the child problems subscale of the PAT would emerge as the primary area of concern, consistent with common referral reasons such as emotional or behavioral difficulties—an expectation anticipated to correspond with elevated SDQ scores in relevant domains; and (2) families experiencing more chronic or severe stress would report greater needs in areas such as parental stress reactions and family structure/resources, potentially aligning with broader or more severe symptom profiles on the SDQ. (3) Our third aim was to examine whether incorporating the PAT and SDQ at the initial point of contact would support more precise, needs-based clinical decision-making, reflected in improved matching between patient psychosocial profiles and symptoms and recommended care pathways. In this context, comprehensive care refers to profile-informed, multimodal treatment recommendations tailored to the child and family’s identified needs, rather than stratification based solely on symptom severity or service intensity. In the context of a clinic based at a tertiary hospital, this would be reflected in an increased proportion of patients receiving comprehensive, multidisciplinary care (i.e., both psychiatric and para-medical interventions) tailored to their specific needs.

## Methods

### Participants

Between January 2024 and January 2025, 941 children and adolescents (542 males, aged 4–18) were referred to the Child and Adolescent Psychiatry Clinic at Sheba Medical Center, Tel Hashomer. The inclusion criteria were parental compliance with the online screening procedure and sufficient mastery of the native language to enable completion of the questionnaires. No formal exclusion criteria were applied; families were included in the analyses if the Parent Assessment Tool (PAT) was fully completed. Demographic data of the children and their parents are presented in Table 1.

**Table 1:** Participant demographic characteristics

Characteristic	*N* = 901, n (%)
Age (years); mean (SD)	10.44 (3.50)
≤ 12 years	622 (69.0%)
> 12 years	279 (31.0%)
Gender	
Male	542 (60.2%)
Female	357 (39.6%)
Other	2 (0.2%)
Religion	
Jewish	875 (97.1%)
Christian	10 (1.1%)
Muslim	7 (0.8%)
Other	9 (1%)
Religious affiliation	
Secular	512 (56.8%)
Traditional	206 (22.9%)
Religious	97 (10.8%)
Ultra-Orthodox	86 (9.5%)
Educational placement	
Mainstream education	563 (62.5%)
Special education	179 (19.9%)
Mainstream education with special support	101 (11.2%)
Dropped out of school	10 (1.1%)
Other	48 (5.3%)
Number of children in the family mean (SD)	2.76 (1.53)
Parent completing the questionnaires	
Mother	787 (87.3%)
Father	111 (12.3%)
Other	3 (0.3%)
Marital status *n* (%)	
Married/ Partners	675 (74.9%)
Divorced or separated	163 (18.1%)
Single	36 (4.0%)
Widowed	11 (1.2%)
Other	16 (1.8%)
Respondent ‘s education level *N* = 898	
Did not complete high school	29 (3.2%)
High school graduate	303 (33.7%)
Bachelor’s/certification student	55 (6.1%)
Bachelor’s/certification graduate	300 (33.4%)
Master’s/PhD student	22 (2.4%)
Master’s/PhD graduate	189 (21.0%)

### Study design/procedure

The study was conducted at a large, multidisciplinary outpatient child and adolescent psychiatry clinic within a tertiary pediatric medical center. The clinic provides comprehensive psychiatric evaluations and a range of specialized interventions, including crisis support, parent-centered treatment, psychodynamic therapy, cognitive behavioral therapy (CBT), trauma-focused care, medical psychology services for emotional distress related to physical illness, and programs targeting learning disabilities, ADHD, and mild neurodevelopmental challenges.

During the study period, completing standardized, parent-reported online screening questionnaires was required as part of the referral process and had to be completed before scheduling an initial psychiatric assessment. This process served as a structured triage designed to guide needs-based, protocol-informed clinical decisions within a tertiary care setting. A senior child psychiatrist (S.T.) reviewed the referral materials and, based on the information provided, determined the appropriate next steps. Screening results guided triage decisions across various factors, including clinical urgency, psychosocial complexity, symptom profile, trauma exposure, and the most likely beneficial intervention type. Families were either placed on the waitlist for an initial psychiatric evaluation or referred to other healthcare, educational, or welfare services when the child’s needs could be better met outside the tertiary outpatient setting.

Notably, screening scores were not used as strict cut-off points for acceptance, exclusion, or automatic pathway assignment. Instead, questionnaire results were viewed as part of a whole, taking into account the overall clinical presentation, developmental context, psychosocial factors, and available treatment resources. This method aimed to improve clinical accuracy and better match patient needs with services, rather than excluding patients solely based on questionnaire scores.

In both the pre- and post-screening phases, final treatment planning and internal allocation to specific therapeutic modalities took place only after an in-person psychiatric evaluation. However, having structured screening information available before intake allowed for earlier identification of clinical complexity and better alignment between referred patients and the specialized services the clinic offers.

Given the clinic’s role as a tertiary care provider, children with relatively mild conditions, such as mild to moderate attention-deficit/hyperactivity disorder without significant comorbidity, were typically referred to community-based mental health, educational, or primary care services better suited to meet these needs.

The Medical Center’s Institutional Review Board (SMC-D-2191-25) approved the study and waived off informed consent for the retrospective analysis of anonymous data that was routinely collected for clinical purposes at the outpatient child and adolescent psychiatric clinic.

### Measures

#### Familial psychosocial risk factors

Psychosocial risk factors were assessed using the Psychosocial Assessment Tool (PAT) 3.0 [26]. The PAT 3.0 comprises seven sub-scales including family structure and resources (8 items; e.g. financial difficulties); social support (4 items; e.g. provision of emotional/financial support); child problems (16 items; e.g. being distracted); sibling problems (16 items; e.g. school or learning difficulties); family problems (15 items; e.g. marital difficulties, separation); parent stress reactions (3 items; e.g. arousal and avoidance); and family beliefs (10 items; e.g. the ability to make good treatment decisions). Subscale scores range from 0.0 to 1.0, based on the proportion of risk-related responses. The total score (0–7) sums the number of risks across all subscales and aligns with the three-tier Pediatric Psychosocial Preventative Health Model [26].

#### Children’s emotional and behavioral functioning

Children’s emotional and behavioral functioning was assessed using the parent-reported Strengths and Difficulties Questionnaire (SDQ) [13]. The SDQ contains four psychopathology subscales: (1) emotional symptoms; (2) behavioral/conduct problems; (3) hyperactivity problems; (4) peer problems, as well as one prosocial functioning subscale. Each subscale has five items, rated on a 0–2 scale. The total difficulties score, based on the four problem subscales, reflects overall behavioral problems, with higher scores indicating greater difficulty. We used an extended version of the SDQ, with an impact supplement. The impact supplement asks whether the respondent thinks the child has a problem, and if so, enquire further about chronicity, distress, social impairment, and burden to others. This provides useful additional information to support treatment planning [27].

#### Children’s exposure to trauma

The Child PTSD Symptom Scale – Parent Report (CPSS-A) is a validated screening measure assessing exposure to traumatic events and PTSD symptom domains in children [15]. In this study, only the exposure section was administered to parents to identify prior exposure to potentially traumatic events and determine a relevant index trauma before the initial psychiatric evaluation. Symptom severity and functional impairment items were not included, as the measure was used exclusively for screening and triage purposes.

### Data analysis

#### Latent profile analysis

To characterize the psychosocial risk factors of our clinical population, we conducted latent profile analysis (LPA) using Mplus 8.8 [28] based on the observed response patterns across seven dimensions of psychosocial risk. Models with one- to six-class solutions were examined. Selection of the optimal class solution was based on the following comparative fit statistics: (1) lower (sample-size adjusted) Bayesian Information Criterion (SA-BIC and BIC) and Akaike Information Criterion (AIC); (2) higher entropy R^2^ values indicating better class separation and values; and (3) significant p-value (< 0.05) of the Vuong–Lo–Mendell–Rubin test (VLMRt), Lo-Mendell-Rubin-Likelihood Ratio Test (LMR-LRt), and the Bootstrap-Likelihood Ratio Test (BLRt). Parsimony and interpretability were also considered when comparing model fit. Full information maximum likelihood estimation was used to account for missing data on LPA indicators. After model comparisons, a three-profile solution was selected as the optimal model, based on both statistical fitness and interpretability.

After identifying the optimal latent profile solution, a three-step approach was conducted to test our second aim: examining the demographic and child emotional-related variables associated with membership in the profiles [29]. This approach employed a classification-error corrected multinomial logistic regression to test differences between classes in demographic variables; child’s gender (1 = girls, 2 = boys), age (in years), parents marital status (1 = single parent, divorced/separated, widow; 2 = married/partners), number of past traumatic events (0–3), and the five distinct scales for the strengths and difficulties questionnaire (SDQ; emotional problems, peer problems, hyperactivity, conduct problems, and prosocial behavior). To evaluate differences in SDQ symptom severity across the identified profiles, one-way ANOVAs with Bonferroni post hoc tests were also conducted.

#### Evaluation of changes in clinical decision-making

To evaluate whether incorporating the PAT, SDQ, and CPSS (A) improved treatment planning (Aim 3), we conducted a pre-post comparison of the proportion of patients receiving comprehensive care. We defined comprehensive care as receiving both psychiatric follow-up and para-medical interventions (e.g., psychological or occupational therapy), according to clinic protocols matching specific patient needs. Data was extracted for all patients seen at the clinic during two three-month periods: October to December 2023 (prior to the implementation of the PAT and SDQ) and October to December 2024 (after implementation). We compared the proportion of patients in each period who received comprehensive care using a chi-square test of independence.

## Results

### Aim 1: feasibility of incorporating the PAT and SDQ into the initial evaluation procedure

A total of 941 parents of children referred to the child and adolescent psychiatry clinic-initiated completion of a set of online questionnaires via the clinic’s website. These included a demographic questionnaire, the Psychosocial Assessment Tool (PAT), the Strengths and Difficulties Questionnaire (SDQ), and the CPSS Checklist.

Of these, 145 parents (15.4%) began the process but did not complete it. Following outreach by the clinic’s administrative staff, 105 parents (11.1%) resumed and completed the questionnaires, 31 parents (3.3%) chose not to proceed with the application process, and 9 parents (0.9%) did not respond to repeated contact attempts. Thus, the final sample consisted of 901 parents who fully completed the screening process. Notably, 84.6% completed the questionnaires without requiring follow-up, and the completion rate increased to 95.7% with minimal outreach attempts, suggesting high feasibility of the online screening procedure in this clinical setting. The sample characteristics are described in Table 1.

### Aim 2: classifying psycho-social risk factors

#### Bivariate analyses

##### Latent profile analysis

Table 2 presents the fit indices for the one- to six-class models. The AIC, BIC and SABIC values decreased consistently from the six-profile model to the 1-class profile model, suggesting improved fit with fewer profiles. All entropy values were excellent across all models (0.94–0.99). According to the LMR, the two-, three-, and five-class solutions had a significant LMR, indicating that these models had a significantly better fit than the model with one less class, but not for the four- and six-profile solutions. Between the two-, three-, and five-profile models, the two- and three-profile solutions had the lowest AIC, BIC, and SABIC. While the two- and three-profile models showed good fit indices, parsimony and interpretability were carefully balanced in the decision making, with the three-class model ultimately selected because it provided the most meaningful and distinct profiles.

**Table 2:** LPA model fit summary

Fit indices							Class 1		Class 2		Class 3		Class 4		Class 5		Class 6	
Model	AIC	BIC	SABIC	Entropy	LMR adjusted *p*-value	BLRt *p*-value	*n*	%	*n*	%	*n*	%	*n*	%	*n*	%	*n*	%
1	-2939.41	-2872.10	-2916.56	-	-	-	-	-	-	-	-	-	-	-	-	-	-	-
2	-3993.47	-3887.70	-3957.57	0.99	0.00	0.00	824	91.05	81	8.95	-	-	-	-	-	-	-	-
3	**-4756.33**	**-4612.09**	**-4707.36**	**0.94**	**0.00**	**0.00**	**712**	**78.9**1	**112**	**12.**65	**77**	**8.**44	**-**	**-**	**-**	**-**	**-**	**-**
4	-5462.38	-5279.67	-5400.36	0.99	*0.16*	0.00	741	81.8	83	9.17	58	6.40	23	2.54	-	-	-	-
5	-6062.51	-5841.34	-5987.43	0.95	0.00	0.00	657	72.61	83	9.17	83	9.25	46	5.08	35	3.86	-	-
6	-6178.40	-5918.77	-6090.27	0.99	0.07	0.00	652	72.05	89	9.82	62	6.88	58	6.40	23	2.54	21	2.28

*AIC*Akaike information criterion; *BIC *Bayesian information criterion; *SABIC* Sample-size adjusted Bayesian information criterion; *LMR* Lo-Mendell-Rubin adjusted likelihood ratio test; *BLRt* Bootstrap likelihood ratio difference test; Bold row was chosen model. *p < .05, **p < .01, ***p < .001

**Fig. 1:**
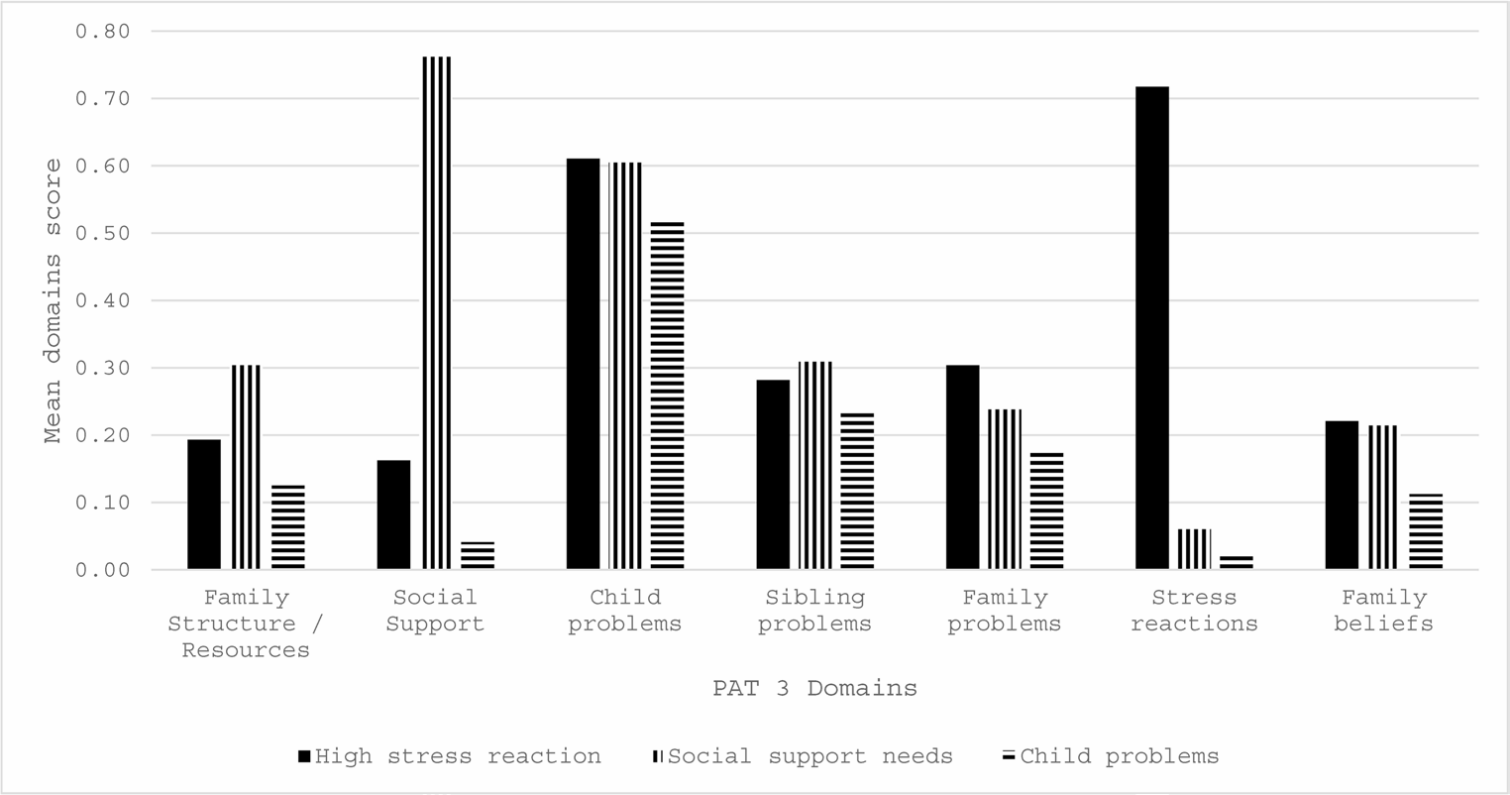
Mean domain PAT scores for each of the 3- profile model of the PAT found by the latent profile analysis (*N* = 901)

Mean domain PAT scores for each of the 3-profile model of the PAT found by the latent profile analysis are indicated by M. The largest latent profile labelled Child Focused Profile (*n* = 716, 79.1%), showed average-low risk across six domains of PAT 3.0 (M < 0.23), except for child problems domain, which had a higher mean of 0.52. The second-largest profile, labelled Low Social Support Profile (*n* = 113, 12.5%), was characterized by elevated risk in social support (M = 0.76) and elevated risk in child problems (M = 0.60). The third latent profile, labelled High Parental Stress Profile (*n* = 77, 8.5%), was distinctly characterized by very high risk in parental stress reactions (M = 0.72) compared to other profiles, and elevated risk in child problems (M = 0.61). See Fig. 1 for a graphical representation of group risk across domains.

#### Predictors of profile membership

In Tables 3, 4, and 5, comparisons between classes in terms of demographic and child emotional-related variables are summarized. Predictors of profile membership were examined via the three-step approach procedure [29]. Correlates of profile membership included in the analysis comprised gender (1 = girls, 2 = boys), child’s age (0–18), parents marital status (1 = single parent, divorced/separated, widow; 2 = married/partners), number of past traumatic events exposure reported (0–3), total difficulties score and the five distinct scales for the strengths and difficulties questionnaire (SDQ); (emotional problems, peer problems, hyperactivity, conduct problems, and prosocial behavior). In Mplus, group comparisons in latent profile models are conducted using a reference class approach to multinomial logistic regression. For interpretability, results are presented as exponentiated regression coefficients (Exp(B)), which represent odds ratios (ORs). The odds ratio reflects how a one-unit increase in the predictor variable affects the odds of membership in the target profile compared to the reference profile. For binary predictors, the OR represents the odds of profile membership for one category relative to the other (e.g., boys vs. girls). For continuous or ordinal predictors (e.g., SDQ subscale scores or number of past traumatic events), the OR indicates the change in odds associated with a one-point increase in the predictor. An OR greater than 1 suggests increased odds of being in the target profile; an OR less than 1 indicates decreased odds. Because the reference group changes across models, the same variable may have different directions of association in different comparisons. For example, an OR of 0.42 for “Past traumatic events” in column (1) means that each additional traumatic event is associated with a 58% decrease in the odds of being in the Child Focused Profile relative to the High Parental Stress Profile, that is, children with more traumas are more likely to belong to the High Parental Stress group. Binary logistic regressions are reported for each pairwise profile comparison. (see Tables 3, 4, and 5).

**Table 3:** Binary logistic regression: profile membership, high stress reaction profile (77) vs. high child problems (712)

Correlates of class membership	B	SE	EXP (B) (C.I 95%)
Child gender (girls = 0, boys = 1)	0.415	0.241	1.514 (0.945–2.428)
Child age (0–18)	0.001	0.005	1.001 (0.995–1.001)
Parents marital status (0 = single parent, divorced/separated, widow; 1 = married/partners)	0.482	0.269	1.620 (0.956–2.746)
Past traumatic events (0–3)	− 0.868	0.152	0.420 (0.312 − 0.565)**
SDQ	− 0.095	0.020	0.909 (0.874 − 0.946)**
Emotional problems	− 0.351	0.057	0.704 (0.630 − 0.786)**
Peer problems	− 0.143	0.051	0.867 (0.785 − 0.957)*
Hyperactivity	− 0.054	0.045	0.948 (0.868–1.035)
Conduct problems	− 0.099	0.050	0.906 (0.821 − 0.999)*
Prosocial behavior	0.028	0.046	1.028 (0.939–1.126)

*SE* Standard error

* Statistically significant difference between classes at *p* < .05. ** Statistically significant difference between classes at *p* < .001

**Table 4:** Binary logistic regression: profile Membership, high child problems (712) vs. high social support needs profile (112)

Correlates of class membership	B	SE	EXP (B) (C.I 95%)
Child gender (girls = 0, boys = 1)	− 0.278	0.205	0.757 (0.507–1.132)
Child age (0–18)	0.002	0.001	1.002 (0.999–1.005)
Parents marital status (0 = single parent, divorced/separated, widow; 1 = married/partners)	-1.363	0.211	0.256 (0.169 − 0.387)
Past traumatic events (0–3)	0.075	0.167	1.078 (0.777–1.497)
SDQ	0.097	0.017	1.102 (1.065–1.140)**
Emotional problems	0.164	0.041	1.178 (1.088–1.276)**
Peer problems	0.179	0.044	1.196 (1.096–1.305)**
Hyperactivity	0.109	0.040	1.115 (1.032–1.205)*
Conduct problems	0.197	0.043	1.217 (1.119–1.324)**
Prosocial behavior	− 0.154	0.039	0.857 (0.794 − 0.926)**

*SE* Standard error

* Statistically significant difference between classes at *p* < .05. ** Statistically significant difference between classes at *p* < .001

**Table 5:** Binary logistic regression: profile membership, high stress reaction profile (77) vs. high social support needs profile (112)

Correlates of class membership	B	SE	EXP (B) (C.I 95%)
Child gender (girls = 0, boys = 1)	0.137	0.297	1.147 (0.641–2.053)
Child age (0–18)	0.003	0.005	1.003 (0.993–1.013)
Parents marital status (0 = single parent, divorced/separated, widow; 1 = married/partners)	− 0.881	0.315	0.415 (0.223 − 0.769)*
Past traumatic events (0–3)	− 0.893	0.230	0.409 (0.261 − 0.642)**
SDQ	0.002	0.023	1.002 (0.957–1.049)
Emotional problems	− 0.194	0.068	0.824 (0.721 − 0.941)*
Peer problems	0.035	0.069	1.036 (0.904–1.186)
Hyperactivity	0.058	0.058	1.060 (0.946–1.187)
Conduct problems	0.095	0.062	1.100 (0.974–1.241)
Prosocial behavior	− 0.123	0.058	0.884 (0.790 – 0.990)*

*SE* Standard error

* Statistically significant difference between classes at *p* < .05. ** Statistically significant difference between classes at *p* < .001

The analysis revealed that age and gender did not significantly differ between any of the identified profiles. A difference in marital status was observed, with children in the Low Social Support Profile having significantly more single/divorced/separated/widow parents compared to the Child Focused Profile and compared to the High Parental Stress Profile. Traumatic events were identified as another significant predictor, with children in the High Parental Stress Profile experiencing more traumatic events compared with the Child Focused Profile and compared to the Low Social Support Profile. Several SDQ subscales were also significantly associated with profile membership. Emotional problems consistently distinguished the High Parental Stress Profile from both other groups, with higher scores predicting greater odds of being in that profile (Tables 3 and 4). When comparing the Low Social Support Profile to the Child Focused Profile (Table 5), higher scores on peer problems, hyperactivity, and conduct problems were associated with increased odds of being in the Low Social Support Profile. Conversely, higher prosocial behavior scores were associated with lower odds of membership in the Low Social Support Profile, distinguishing it from both other profiles. These findings suggest distinct patterns of emotional and behavioral difficulties across profiles, with emotional distress characterizing the High Parental Stress Profile and broader behavioral challenges marking the Low Social Support Profile.

Table 6 presents SDQ subscale scores across the three latent profiles using one-way ANOVA with Bonferroni post hoc tests. Statistically significant differences were observed across all SDQ domains. The High Parental Stress Profile (Profile C) had the highest scores for emotional problems (M = 7.49) and total difficulties (M = 22.29), indicating pronounced internalizing symptoms and overall distress. In contrast, the Low Social Support Profile (Profile B) showed the highest levels of peer problems (M = 4.59), hyperactivity (M = 6.71), and conduct problems (M = 4.60), as well as the lowest prosocial behavior score (M = 5.60), suggesting substantial behavioral difficulties and possible social withdrawal or limited empathy. The Child Focused Profile (Profile A) had significantly lower scores across all SDQ difficulty subscales compared to both other profiles, indicating relatively fewer psychosocial concerns. These findings highlight distinct clinical patterns among the profiles, reinforcing their interpretability and relevance for tailoring interventions.

**Table 6:** Onaway ANOVA with bonferroni post hoc tests, SDQ scale scores across latent profile classes

SDQ scale | Profile	High child problems A	High social support needs B	High stress reaction C	Sig
SDQ total	18.39 (6.63)	22.36 (6.33)	22.29 (6.47)	< 0.001 ^a^
Emotional problems	5.34 (2.70)	6.46 (2.46)	7.49 (2.16)	< 0.001 ^b^
Peer problems	3.63 (2.32)	4.59 (2.04)	4.43 (2.31)	< 0.001 ^c^
Hyperactivity	5.93 (2.76)	6.71 (2.55)	6.32 (2.60)	< 0.05 ^d^
Conduct problems	3.48 (2.31)	4.60 (2.40)	4.04 (2.50)	< 0.001 ^e^
Prosocial behavior	6.63 (2.53)	5.60 (2.46)	6.44 (2.86)	< 0.001 ^f^

^a.^ A < B**, A < C**

^b.^ A < B**, A < C**, B < C*

^c.^ A < B**, A < C**

^d.^ A < B**

^e.^ A < B**

^f.^ A > B**

### Aim 3: evaluation of clinical decision-making precision

From October to December 2024, following the implementation of the screening tools, 927 patients attended the clinic, of whom 761 (82.1%) received comprehensive care, defined as combined psychiatric and psychotherapeutic intervention. During the same period in 2023, before implementation, 1,008 patients attended, with 781 (77.5%) receiving comparable care. A chi-square test of independence revealed a statistically significant increase in the proportion of patients receiving comprehensive care after the introduction of the PAT and SDQ, χ²(1, *N* = 1935) = 6.35, *p* = .012. During the post-implementation period, a higher proportion of patients entering the tertiary outpatient clinic were assigned to multi-modal care pathways, while patients with less complex presentations were more often referred to alternative services before intake.

Accordingly, the distribution of care pathways changed between the pre- and post-implementation periods, with a greater proportion of patients receiving comprehensive care after the introduction of structured screening. This indicates that the screening tools may have helped produce more personalized treatment planning.

## Discussion

This study classified patients in child and adolescent psychiatric outpatient care based on parent-reported psychosocial risk and protective factors (PAT) and examined their links to symptom severity (SDQ, CPSS-A). Findings suggest that this dual-assessment process not only facilitates the identification of symptom severity and psychosocial risk but also enables the classification of patients into meaningful profiles that can inform both triage and individualized treatment planning.

The PAT LPA identified three distinct psychosocial profiles. A summary of the demographic, psychosocial, behavioral and trauma exposure characteristics of these three PAT-based profiles is presented in Tables 3, 4, and 5. The largest, the Child-Focused Profile (80% of participants), reflects referrals driven mainly by child-centered difficulties, with symptoms in one domain (e.g., hyperactivity) often affecting others (e.g., peer relationships, conduct). From a clinical and service-planning perspective, this pattern suggests that triage and treatment should prioritize interventions targeting the child’s presenting difficulties, while maintaining sensitivity to the broader family context. Accordingly, treatment planning for this profile is likely to center on individualized, child-focused interventions, such as individual psychotherapy, behavioral approaches, school-based supports, and developmentally informed parental guidance, tailored to the child’s specific clinical needs. Within this broader intervention framework, cognitive-behavioral therapy (CBT) represents one commonly utilized evidence-based approach, particularly in cases where parental engagement and motivation support sustained therapeutic work [30].

The High Parental Stress Profile, though smaller in size (9%), is clinically significant. Children in this group were exposed to more traumatic events and showed the highest emotional and total difficulties, consistent with research linking parental stress and trauma to heightened child mental health difficulties and disrupted family functioning [31]. The strong association between trauma and this profile highlights the need for trauma-informed, family-centered care in outpatient psychiatry. Notably, this group’s severe SDQ scores show how parental stress and trauma amplify children’s psychiatric symptoms [32], emphasizing the importance of assessing parental stress to tailor interventions to both child and family needs [33]. For families characterized by the High Parental Stress Profile, interventions may reasonably prioritize parent- and family-focused approaches aimed at reducing caregiver distress and improving parent–child interactions [34], with evidence-based models such as Dialectical Behavior Therapy (DBT) [35], Supportive Parenting for Anxious Childhood Emotions (SPACE) [36], and dyadic interventions like Parent–Child Interaction Therapy (PCIT) [30] serving as illustrative examples of frameworks that target parental regulation and relational processes.

The Low Social Support Profile (13%) marked by elevated risk in social support and significant child problems, aligns with a substantial body of research emphasizing the importance of social support for youth well-being [37]. Children in this group had more peer problems, hyperactivity, and conduct problems compared to the Child Focused Profile, alongside the lowest prosocial behavior. Compared to the High Parental Stress group, which was defined by emotional distress, this profile was characterized by behavioral and social difficulties, Low support not only strains parenting capacity but also increases child vulnerability, particularly in single-parent households where isolation is common [38, 39]. for families in the Low Social Support Profile, early emphasis may be placed on addressing contextual and systemic stressors, including linkage to social, educational, and welfare resources, as a complement to clinical care [40].

Psychosocial profiles support shifting from uniform waitlists to profile-informed, needs-based triage in child psychiatry, guiding not only the intensity but also the type of recommended interventions. The High Parental Stress Profile should be prioritized to prevent intergenerational stress transmission [32], including parental psychiatric assessment, trauma evaluation and stress-reduction interventions. The Low Social Support Profile requires early connection to social services, community resources and group interventions to reduce isolation and strengthen resilience. The Child-Focused Profile can benefit from targeted child-centered interventions for emotional and behavioral issues. Profile-guided triage improves resource allocation and ensures timely care for high-risk families. Integrating the PAT, SDQ, and CPSS-A tools increased comprehensive care delivery, suggesting these instruments enhance need-based treatment planning. While causality cannot be confirmed, the identification of risk profiles enables individualized planning, consistent with contemporary child mental health care [41].

Our findings align with a prior single-site study that examined use of the PAT during initial psychiatric evaluation and identified three predominant domains of psychosocial stressors: child symptom burden, parental stress reactions, and family structure and support needs [25]. Although that study demonstrated improved treatment adherence among families receiving PAT-informed intensive case management, the screening was administered only after prolonged wait-list periods and therefore did not inform initial triage or intake decisions. In contrast, the present study implemented the PAT as part of an integrative screening battery at the point of referral, prior to clinical intake. While we similarly identified three distinct psychosocial profiles that closely map onto these domains of need, we did not assess downstream referral pathways or treatment outcomes. Accordingly, our findings represent a conceptual replication of the psychosocial risk structure identified in earlier work, while extending screening upstream to the intake phase, highlighting the need for future studies to evaluate whether early, profile-informed screening can yield comparable improvements in treatment engagement and clinical outcomes.

Our study has several limitations. First, although the high screening completion rate (95.7%) suggests strong engagement with the screening process, acceptability was not directly assessed through satisfaction or perceived burden measures and should be examined in future studies. Second, the intentional partial administration of the CPSS-5, limited to trauma exposure screening, constrains conclusions regarding post-traumatic symptom severity and functional impairment, highlighting the need for subsequent trauma-focused assessment among children who screen positive for exposure. Third, while three psychosocial profiles were identified, the clinical utility of this classification could not be evaluated, as data on subsequent referral pathways and treatment outcomes were not collected; thus, it remains unclear whether this profiling approach improves wait times, treatment adherence, or overall effectiveness. Fourth, the screening procedure relied exclusively on parent-report measures, without concurrent youth self-report at the initial referral stage. This design choice was consistent with the study’s emphasis on feasibility and minimal burden during first contact, and aligns with prior screening frameworks using tools such as the PAT [14, 16] and SDQ [21, 42, 43], as well as trauma screening models recommending brief parent-report assessment at initial triage [24]. Nonetheless, the absence of youth self-report may have reduced sensitivity to internalizing or trauma-related symptoms, and future studies should examine whether incorporating earlier multi-informant screening enhances triage accuracy and downstream outcomes. Finally, the single-clinic sample limits generalizability, as the identified profiles may reflect characteristics of this specific population. Nonetheless, the analytic approach used, linking psychosocial risk and symptom data to identify subgroups, can be applied broadly. We encourage replication in other clinics to reveal population-specific profiles and guide more tailored service planning and resource allocation.

Despite these limitations, the study is notable for its large sample size (*N* = 901) and the high completion rate of the PAT, SDQ and CPSS-5 (A) among referred families, underscoring the feasibility of integrating these tools into pediatric psychiatric outpatient settings. Future studies should include longitudinal follow-up to examine the impact of screening on waitlist times, treatment adherence, and clinical outcomes for each of the identified profiles. Including child self-reports and teacher assessments would further strengthen the robustness of the classification model and its predictive value.

## Data Availability

The datasets generated and/or analyzed during the current study are not publicly available due to patient confidentiality but are available from the corresponding author on reasonable request.
